# 

**Published:** 1881-07

**Authors:** 


### CONTENTS.

PAGE.

The Proceedings of the International Hahnemannian Association; The First Meeting, . . . . . . . . . . 273
Address of the President on the Philosophy of Homœopathy. P. P. Wells, M. D., Brooklyn, N. Y., . . . . . . . . 277
Drug Proving. Ad. Lippe, M. D., Philadelphia, . . . . . 287
Proving of Amm. Carb. Leila A. Rendell, M. D., New Haven, Conn., 292
Conservative Surgery. H. I. Ostrom, M. D., New York, . . . 305
Abnormalities of Labor. L. B. Wells, M. D., Utica, N. Y., . . . 310
Diseases of Infants. C. Lippe, M. D., New York, . . . . . 312
Similia Similibus Curantur. Geo. F. Foote, M. D., Stamford, Conn., . 314
Magna est Veritas, et Prævalebit. Ad. Fellger, M. D., Philadelphia, . 316
Pathological Prescribing: "A Science Falsely So-called." E. J. Lee, M. D., Philadelphia, . . . . . . . . . 319

*CLINICAL BUREAU:*

Cases Cured by Metals. Wm. P. Wesselhœft, M. D., Boston, . . . 326
The False and the True: a Warning. Geo. H Clark, M. D., Philadelphia, . . . . . . . . . . . . 332
Lyssin in Hydrophobia; Euthanasia from the Simillimum. E. W. Berridge, M. D., London, . . . . . . . . . . 334
Some Clinical Cases. E. Rushmore, M D., Plainfield, N. J., . . . 338
Superiority of High Potencies over Low in the Treatment of Worst Forms of Disease. R. R. Gregg, M. D., Buffalo, N. Y. . . . 341
Clinical Cases. John F. Miller, M. D., Newark, N. J . . . . 348
Legitimate Homœopathy. C. Pearson, M. D., Washington, D. C. . . 351

